# Critical analysis of the hypothesized *SNHG1*/*miR-195-5p*/*YAP1* axis

**DOI:** 10.1007/s10142-022-00930-z

**Published:** 2022-12-17

**Authors:** Steven P. Zielske, Frank C. Cackowski

**Affiliations:** Department of Oncology, Wayne State University and Karmanos Cancer Institute, Detroit, MI 48201 USA

How lncRNAs such as *SNHG1* are integrated into cellular pathways to regulate, or be a regulatory component of, cellular processes is a question yet to be completely answered. A potential mechanism by which *SNHG1* may exert its effect is through the so-called sponge effect. In this mechanism, *SNHG1* (or other lncRNAs) is thought to bind miRNAs as a molecular sponge in a sequence-specific manner and inhibit their ability to bind and inhibit translation of a specific mRNA. The recent paper by Cheng et al. (Cheng et al. 2022) makes just these connections, in this case, between *SNHG1*, *miRNA-195-5p*, and the Hippo pathway gene, *YAP1*. In their proposed model, *miRNA-195-5p* binds to, and causes degradation of, *YAP1* mRNA. *MiRNA-195-5p* is in turn modulated through the sponging activity of *SNHG1.* These interactions theoretically occur through a region of 7 nucleotide complementarity. Thus, YAP1 target protein expression can be modulated through a complex interplay involving the degree of *SNHG1* and *miR-195-5p* expression. High *SNHG1* should lead to greater sponging of *miR-195-5p*, and result in high *YAP1* mRNA and protein levels, and conversely, low *SNHG1* should lead to less sponging of *miR-195-5p*, and therefore low *YAP1* mRNA and protein levels.

However, we would like to call attention to some aspects of the published study which are common in the field overall and to offer a counterpoint to the conclusions drawn. In the human genome, there are possibly 183,000 occurrences of the 7 nucleotide sequence being suggested to confer specificity in the binding of *SNHG1* to *miR-195-5p* and, subsequently, *YAP1* (calculated as the 3 billion bp human genome divided by the frequency of random appearance of the given 7 nt sequence – 4^7^). Within protein-coding regions, which are estimated to be 1.5% of the genome, there may be 2746 (4.5 × 10^7^/4^7^) occurrences of this sequence. Indeed, miRNA target prediction websites (miRDB, miRDB.org and TargetScanHuman, targetscan.org) predict 1419–1508 transcript targets of *miR-195-5p* based on binding in the 3’ UTR. Interestingly, and relevant to the Cheng et al. study, *miR-195-5p* is not only predicted to interact with *YAP1*, but also *LATS2* through the exact same sequence. *LATS2* is a member of the Hippo pathway and a direct negative regulator of *YAP1*. This significantly increases the complexity of the potential effects of *miR-195-5p* on Hippo pathway signaling and profoundly affects the interpretation of the results of the Cheng study, which focuses exclusively on *YAP1*. Still, whether the biological effects observed after modulation of *SNHG1* or *miR-195-5p* occur through the Hippo pathway, or one of the other predicted target genes, is unknown.

Another concern of the work of Cheng et al. is that their published sequence for *SNHG1* does not appear in the *SNHG1* processed transcript, but the entire 21 nt sequence is in an *SNHG1* intron. This means that the *SNHG1* sequence used in their reporter studies cannot act as a sponge for *miR-195-5p*. However, the correct *SNHG1* sequence does have an 8 nt span of complementarity to *miR-195-5p* and has the potential to sponge miR-195-5p (Fig. 1). The resulting impact on their reported studies is that the luciferase reporter experiments using their *SNHG1* sequence insert are not utilizing the correct sequences and are thus invalid.

**Fig. 1:**
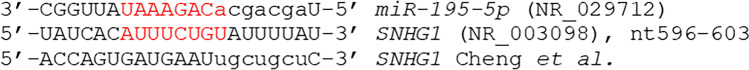
RNA sequences of *miR-195-5p* and *SNHG1* from the National Center for Biotechnology Information and the RNA sequence for *SNHG1* given by Cheng et al. Red letters indicate complementarity between the official gene sequences. Lowercase letters indicate complementarity between Cheng’s *SNHG1* sequence and *miRNA-195-5p*

The observations made after *SNHG1* knockdown may be valid, but linking those phenotypes specifically to *YAP1* through *miR-195-5p* is difficult. A connection between *SNHG1* and *YAP1* in squamous cell carcinoma was previously claimed in 2020 through *miR-375*. This study was subsequently retracted (Gao et al. 2020). Likewise, the first report of a connection between *SNHG1* and *miR-195* has also been retracted (Zhang et al. 2021). Others have reported the possible connection between *SNHG1* and *miR-195*, but with many target genes, including *CCND1*, *PDCD4*, *NEK2*, and *CDC42* (Li et al. 2019; Huang et al. 2019; Ji et al. 2020; Chen et al. 2021). Few of these reports show a direct effect on the target mRNA upon *SNHG1* knockdown but rely on luciferase reporter constructs to show sequence-specific activity between *SNHG1* and the reported miRNA, and then between the miRNA and the target mRNA. However, in the luciferase reporter constructs, it is only the identified complementary sequence that is in the reporter. This system is designed to work but does not reliably recapitulate the cell biology.

Likewise, the current study does not show a change in endogenous *YAP1* expression upon *SNHG1* knockdown and relies on the circumstantial evidence of a luciferase reporter assay. Indeed, our data in prostate cancer indicate a modest increase in *YAP1* expression following *SNHG1* knockdown (Fig. 2a). Furthermore, when we compare cellular proliferation after *SNHG1* and *YAP1* knockdown, we see a greater magnitude decrease in proliferation after *SNHG1* knockdown than *YAP1* knockdown, even though the knockdown of *YAP1* (90%) was significantly better than *SNHG1* (60%) (Fig. 2b). If *YAP1* were more proximal in the pathway than *SNHG1*, then we would expect a greater effect with modulation of *YAP1* level than *SNHG1* level.

**Fig. 2:**
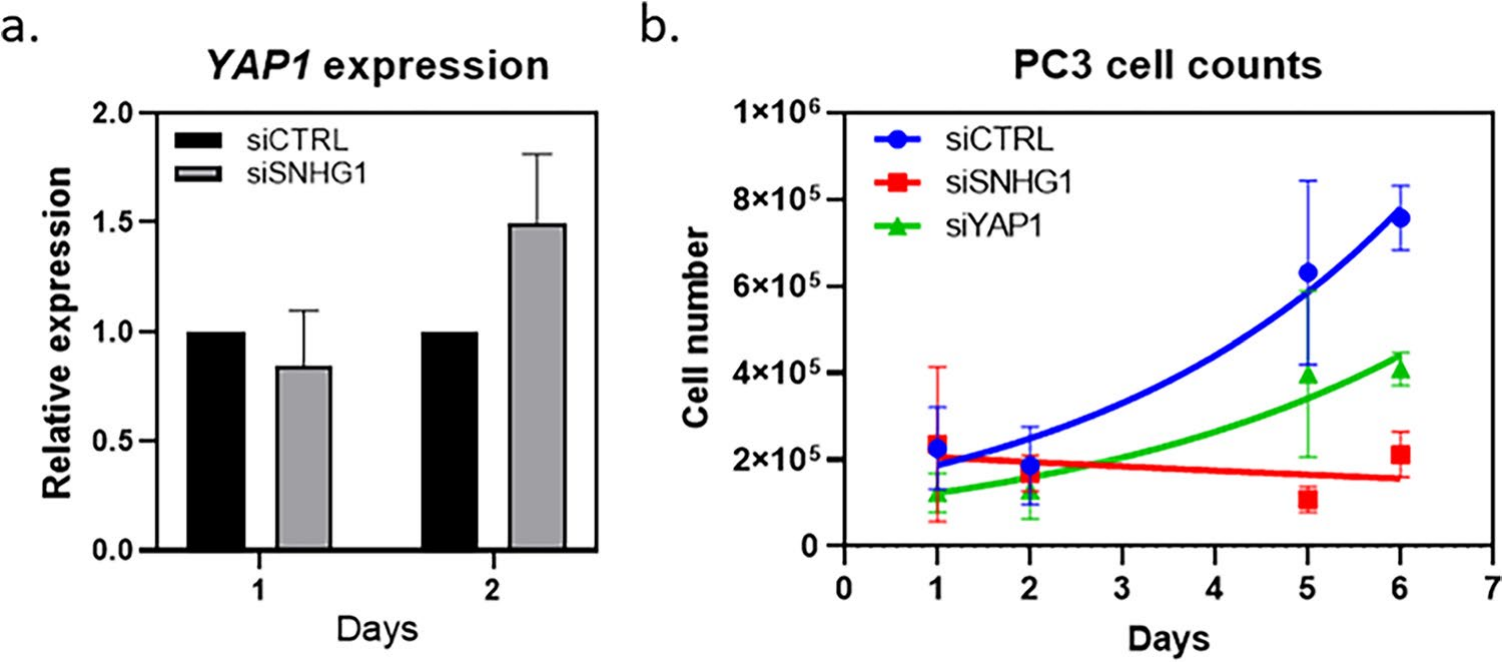
Results of *SNHG1* or *YAP1* knockdown. **a** *SNHG1* knockdown in PC3 prostate cancer cells results in a modest increase in *YAP1* expression 2 days after siRNA transfection. **b** *YAP1* knockdown in PC3 cells results in slowed growth, but *SNHG1* knockdown results in complete growth arrest

Overall, we point out that the proposed mechanism and findings of Cheng et al. lack specificity due to the short nucleotide region of complementarity between *miR-195-5p* and *YAP1*, and some of the data is questionable because of the use of an incorrect sequence for *SNHG1.* In addition, their findings rely on luciferase reporter assays rather than measurement of endogenous RNA. We have observed findings opposite to what their model predicts, although admittedly in cell lines from a different cancer. Finally, we show that *miR-195-5p* is also predicted to interact and potentially regulate *LATS2*, a negative regulator of *YAP1*. These issues with the work by Cheng et al. are consistent with problems observed in the *SNHG1* literature overall, including reliance on luciferase reporter constructs and incorrect sequences. We also note a significant number of retractions in this field due to fraudulent data. Therefore, we urge caution in interpreting the results of Cheng and colleagues and the *SNHG1* literature overall.

## Data Availability

The datasets generated during and/or analyzed during the current study are available from the corresponding author on reasonable request.
